# CD271+, CXCR7+, CXCR4+, and CD133+ Stem/Progenitor Cells and Clinical Characteristics of Acute Ischemic Stroke Patients

**DOI:** 10.1007/s12017-018-8494-x

**Published:** 2018-05-09

**Authors:** Anna Gójska-Grymajło, Maciej Zieliński, Dariusz Gąsecki, Kamil Kowalczyk, Mariusz Kwarciany, Barbara Seroczyńska, Walenty M. Nyka

**Affiliations:** 1 Department of Adult Neurology, Medical University of Gdańsk, University Clinical Center, Dębinki 7, 80-211 Gdańsk, Poland; 2 Department of Clinical Immunology and Transplantology, Medical University of Gdańsk, Gdańsk, Poland; 3 Department of Medical Laboratory Diagnostics and Bank of Frozen Tissues and Genetic Specimens, Medical University of Gdańsk, Gdańsk, Poland

**Keywords:** Stem cells, Ischemic stroke, CXCR7, CD271, CXCR4, CD133

## Abstract

Ischemic stroke causes mobilization of various groups of progenitor cells from bone marrow to bloodstream and this correlates with the neurological status of stroke patients. The goal of our study was to identify the activity of chosen progenitor/stem cells in the peripheral blood of acute ischemic stroke patients in the first 7 days after the incident, through associations between the levels of the cells and clinical features of the patients. Thirty-three acute ischemic stroke patients and 15 non-stroke control subjects had their venous blood collected repeatedly in order to assess the levels of the CD45–CD34 + CD271+, the CD45–CD34 + CXCR4+, the CD45–CD34 + CXCR7+, and the CD45–CD34 + CD133+ stem/progenitor cells by means of flow cytometry. The patients underwent repeated neurological and clinical assessments, pulse wave velocity (PWV) assessment on day 5, and MRI on day 1 and 5 ± 2. The levels of the CD45–CD34 + CXCR7+ and the CD45–CD34 + CD271+ cells were lower in the stroke patients compared with the control subjects. Only the CD45–CD34 + CD271+ cells correlated positively with lesion volume in the second MRI. The levels of the CD45–CD34 + CD133+ cells on day 2 correlated negatively with PWV and NIHSS score on day 9. The patients whose PWV was above 10 m/s had significantly higher levels of the CD45–CD34 + CXCR4+ and the CD45–CD34 + CXCR7+ cells on day 1 than those with PWV below 10 m/s. This study discovers possible activity of the CD45–CD34 + CD271+ progenitor/stem cells during the first 7 days after ischemic stroke, suggests associations of the CD45–CD34 + CD133+ cells with the neurological status of stroke patients, and some activity of the CD45–CD34 + CD133+, the CD45–CD34 + CXCR4+, and the CD45–CD34 + CXCR7+ progenitor/stem cells in the process of arterial remodeling.

**Electronic supplementary material:**

The online version of this article (10.1007/s12017-018-8494-x) contains supplementary material, which is available to authorized users.

## Introduction

Cerebral stroke causes an efflux of various groups of progenitor and stem cells from bone marrow to bloodstream and the levels of these cells correlate with the neurological status of stroke patients (Dunac et al. 2007; Gójska-Grymajło et al. 2012; Hennemann et al. 2008; Paczkowska et al. 2005; Sobrino et al. 2007). It is the paracrine effect of the adult stem cells that seems to play the major role in rescuing cerebral ischemic tissue (Beer et al. 2016; Madhavan and Collier 2010; Shimada and Spees 2011). Numerous factors produced by the injured tissue attract progenitor/stem cells that in turn secrete a variety of trophic factors and extracellular vesicles. The ischemic niche interactions seem to be very subtle and complex and some of the factors may evoke opposed actions (Bang et al. 2016; Blanchet et al. 2012; Mirabelli-Badenier et al. 2011).

There are still too few studies unraveling the complexity of the interactions of the cerebral ischemic niche in humans. Only a few of them consider the influence of stem cells dynamics, lesion volume, and co-morbidities, even though such correlations had been found long ago (Bogoslovsky et al. 2010; Carvalho et al. 2015; Dunac et al. 2007; Fadini et al. 2006; Gojska-Grymajlo et al. 2012).

It seems plausible that tracing down the subgroups of progenitor/stem cells specifically evoked from bone marrow by brain ischemia could result in finding the optimal subgroup of cells to be used in cell or secretome therapies.

The goal of our study was to trace down possible associations of the selected subgroups of progenitor/stem cells in the peripheral blood of acute ischemic stroke patients with clinical features (neurological and functional status, co-morbidities, ischemic lesion volume, arterial stiffness, and laboratory parameters).

We have chosen the CD45–CD34 + CD133+ endothelial progenitor cells that have been most commonly studied in cerebral stroke patients. Initially, these cells were suspected to have the biggest regenerative potential for ischemic brain (Bakondi et al. 2009). This group of cells is also interesting for their possible correlations with arterial stiffness, that were proved to be significant in a study on patients with hypertension (Marketou et al. 2014).

Secondly, we have chosen the CD45–CD34 + CD271+ cells, with CD271+, which is a surface marker indicative of a subset of mesenchymal progenitor cells with high proliferative, clonogenic, and multipotential differentiation ability in adult bone marrow and adipose tissue (Quirici et al. 2010). CD271 is also an important antigen of melanoma and osteosarcoma cells that indicates high self-renewal rates, drug resistance, and tumorigenicity. Its presence on melanoma cells indicates special aptitude for brain metastasis (Boiko et al. 2010; Guo et al. 2014; Tian et al. 2014). The CD271+ cells were proved to be mobilized early after myocardial infarction (Iso et al. 2012). There have been no studies concerning possible mobilization of this group of cells in patients with ischemic cerebral stroke so far.

The CD45–CD34 + CXCR4+ progenitor/stem cells constitute another group. CXCR4 is a major player of the SDF-1α–CXCR4/CXCR7 axis and determines the migration of cells towards the ischemic lesion. In our previous work (Gójska-Grymajło et al. 2012), we have shown that the CD45–C34+, CD45–CD34 + CXCR4+, and the CD45–CXCR4+ cells are present in the peripheral blood of ischemic stroke patients in low numbers, but their levels and dynamics seem to correlate with the functional status of the patients.

Finally, we have chosen the CD45–CD34 + CXCR7 progenitor/stem cells, with CXCR7 receptor that seems to be crucial for the SDF-1α–CXCR4/CXCR7 axis. It is expressed at very low levels (approx. 3–6%) in normal human CD34+ cells isolated from bone marrow, umbilical cord blood, and mobilized peripheral blood (Tarnowski et al. 2010). Its expression is increased in inflammatory, hypoxic, and neoplastic processes and its presence on the surface of a cell modifies CXCR4 functions (Sanchez-Martin et al. 2013). In the last 2 years, it has emerged as a potentially important receptor for therapeutic interventions in various neoplasms, myocardial infarction, autoimmune CNS diseases, or lung fibrosis (Bao et al. 2016; Cao et al. 2016; Demir et al. 2017; Deng et al. 2017; Hao et al. 2017; Huang et al. 2017). Interestingly, CXCR7 promoted migration of bone marrow mesenchymal stem cells towards SDF-1 gradient in a cerebral ischemia–reperfusion rat hippocampus model (Wang et al. 2014). However, there have been no studies concerning stem/progenitor cells with this antigen in ischemic stroke patients so far.

## Methods

### Study Population

Thirty-three patients (14 women and 19 men; mean age 66.8 years) with acute ischemic stroke hospitalized in the Department of Adult Neurology of the Medical University of Gdańsk, Poland, and 15 control subjects (six women and nine men; mean age 62.2 years) were included in the study. The patients were included in the study within 17.28 ± 9.5 h after the incidence of ischemic stroke.

In the stroke subjects, contraindications for the inclusion in the study were as follows: previous stroke (ischemic and/or hemorrhagic), pregnancy, pre-existing dependency (modified Rankin Scale score > 2 points), contraindications for magnetic resonance imaging (MRI) study, and atrial fibrillation on admission.

In the control group, a contraindication for inclusion was cerebral stroke in the past.

The study was approved by the local ethics committee. All the patients and the control subjects provided written, informed consent for the involvement in the study.

### Blood Collection and Clinical Assessment

Peripheral blood was collected into EDTA-coated tubes (Bection Dickinson Vacutainer) from each patient, shortly after inclusion in the study (day 1) as well as on day 2 and 7. In the case of the patients that received thrombolytic treatment, the first collection was performed after the treatment. Blood from the control group was collected once, incidentally.

On day 1, 2, and 9, patients were assessed using the National Institutes of Health Stroke Scale (NIHSS). Modified Rankin Scale (mRS) was used to assess patients’ functional status on admission (pre-mRS) and on day 9. Additionally, patients were classified into four subgroups according to the Oxfordshire Community Stroke Project definition: total anterior circulations infarcts (TACI), partial anterior circulation infarcts (PACI), posterior circulation infarcts (POCI), and lacunar infarcts (LACI) and into five subgroups according to the etiological TOAST (trial of ORG 10172 in acute stroke treatment) classification: large-artery atherosclerosis, cardioembolism, small-vessel occlusion, undetermined etiology, and other determined etiology (Table 1).

**Table 1:** Baseline characteristics of the stroke and the control group

Variables	Stroke group	Control group	*p* value^*^
Age, mean ± SD [years]	66.8 ± 12.3	62.2 ± 3.7	0.09
Sex, male	19 (57.6%)	9 (60%)	0.84
Hypertension	30 (90.9%)	15 (100%)	0.22
Atrial fibrillation			
Paroxysmal	5 (15.2%)	0 (0.0%)	0.11
Chronic	0 (0.0%)	1 (6.7%)	0.13
Diabetes mellitus			
Oral drugs	9 (27.3%)	1 (6.7%)	0.10
Insulin-dependent	4 (12.1%)	5 (33.3%)	0.08
Coronary artery disease	9 (27.3%)	4 (26.7%)	0.90
Myocardial infarction	5 (15.2%)	3 (20.0%)	0.67
Hyperlipidemia	24 (72.7%)	5 (33.3%)	**0.0097**
Obesity	18 (54.5%)	2 (12.5%)	**0.003**
Smoking			
Present	11 (33.3%)	1 (6.7%)	**0.027**
Past	19 (57.6%)	5 (33.3%)	**0.04**
Alcohol abuse			
Present	1 (3.0%)	1 (6.7%)	0.48
Past	4 (12.1%)	1 (6.7%)	0.48
rtPA treatment	11 (33.3%)		
OCSP classification			
TACI	4 (12.1%)		
PACI	15 (45.5%)		
POCI	8 (24.2%)		
LACI	6 (18.2%)		
TOAST classification			
Large-artery atherosclerosis	7 (21.2%)		
Cardioembolism	6 (18.2%)		
Small-vessel occlusion	4 (12.1%)		
Undetermined etiology	15 (45.5%)		
Other determined etiology	1 (VA dissection 3.0%)		
NIHSS (median; range)			
Day 1	5; 1–23		
Day 9	2; 0–11		
mRS (median; range)			
Day 1 (pre-mRS)	0; 0–2		
Day 9	2; 0–4		

Statistically significant values (*p* < 0.05) are given in bold

*OCSP* Oxfordshire Community Stroke Project; *TACI* total anterior circulations infarcts; *PACI* partial anterior circulation infarcts; *POCI* posterior circulation infarcts; *LACI* lacunar infarcts. TOAST, trial of ORG 10172 in acute stroke treatment classification; *rtPA* recombinant tissue plasminogen activator

^*^The χ^2^ test was used to compare sex and the cerebrovascular risk factors between the stroke group and the controls. The groups differed significantly in terms of hyperlipidemia, obesity, and smoking. The Mann–Whitney U test was used to compare age between the patients and the control subjects

### Flow Cytometry Analysis

For each sample, 100 µl of peripheral EDTA blood was stained with monoclonal antibody cocktail: CXCR7 (FITC; clone 358426; R&D Systems), CXCR4 (PE-Cyanine7; clone 12G5; eBioscience), CD271 (PE, clone ME20.4-1.H4, Miltenyi Biotec), CD133/2 (APC, clone 293C3, Miltenyi Biotec), CD34 (eFluor450; clone 4H11; eBioscience), and CD45 (Krome Orange; clone J33; Beckman Coulter). Then, samples were lysed using lyse no-wash method with Immunoprep Reagent System and TQ-Prep Workstation (Beckman Coulter). The readout was done with Beckman Coulter Navios Flow Cytometer and a total count of 100,000 cells was acquired.

Samples were analyzed according to the SSc signal and CD45 expression and then the gate was put on the CD45 low to negative events (CD45 negative gate). Subsequently, the CD45 negative cells were analyzed as SSc low and the CD34 positive cells (CD45 negative/CD34 positive gate). Finally, expression of CXCR7, CXCR4, CD271, and CD133/2 was measured, in regard to the CD45 negative/the CD34 positive gate. The values were reported as mean fluorescence intensity (MFI). The analysis was done with Kaluza Software (Beckman Coulter).

### MRI Study

Each patient underwent the first magnetic resonance imaging scan (MRI 1) on the day of admission. The control MRI—(MRI 2) was performed on day 5 (± 2) after stroke onset. The protocol of the MRI 1 included diffusion-weighted imaging (DWI), fluid attenuation inversion recovery (FLAIR), susceptibility-weighted imaging (SWI), and time-of-flight angiography (TOF). The MRI 2 protocol, apart from the above-mentioned, included T1- and T2-weighted imaging.

Lesion volume was estimated semi-automatically (Brain Analyzer). Lesion growth was estimated by subtracting DWI-MRI1 from DWI-MRI 2 lesion volume.

### Pulse Wave Velocity

Pulse wave velocity (PWV), a marker of arterial stiffness, was measured on day 5 using applanation tonometry (AT) with a SphygmoCor device (Atcor, Sydney, Australia) by one of two physicians trained specifically in this technique (MK, KK).

Repeatability and reproducibility of applanation tonometry measurements were calculated using two-way mixed single measures with an intraclass correlation coefficient (ICC) for absolute agreement: inter- and intra-observer reliabilities, calculated by the ICC, were 0.86 and 0.96, respectively (thus indicating excellent reproducibility and repeatability). All the measurements were performed with standardized conditions. These conditions, recommended by expert consensus, had been described previously for other research projects by our group (Gąsecki et al. 2012; Kwarciany et al. 2016; Laurent et al. 2006). Briefly, the applanation probe was positioned on the radial artery, and optimal applanation was obtained through visual inspection and through the device’s built-in quality control indices. Radial waveforms were calibrated using the average brachial systolic blood pressure and diastolic blood pressure (DBP) measured before and after the applanation. The central aortic waveform was calculated with the device software using a generalized transfer function, and blood pressure values were derived from this curve. Following the central augmentation index (cAIx) measurement, the cAIx at a heart rate of 75 beats per minute was calculated by the software. The procedure was repeated on the common carotid artery, and the calibration was made using DBP and mean blood pressure obtained from the radial tracings. Immediately afterwards, applanation was performed on the femoral artery, and pulse transit times from concomitant electrocardiogram were calculated using the intersecting tangent. The real carotid-femoral pulse wave velocity (CF-PWV) was measured in this study. It means that the distance used in the measurement was obtained after applying a 0.8 scaling factor to the direct distance between the two recorded sites (the carotid and the femoral arteries).

### Laboratory Parameters

We took into consideration C-reactive protein (CRP) assessed routinely on admission in each stroke patient and the highest count during hospitalization, which reflects the intensity of various inflammatory processes during the acute phase of stroke that could influence the efflux of the stem/progenitor cells into the peripheral blood. Hemoglobin, hematocrit, and white blood count were taken into consideration to exclude evident hematologic neoplasms and serious inflammatory reactions. Finally, we included lipid profiles in our assessments (low-density lipoprotein (LDL), high-density lipoprotein (HDL), and triglycerides—results obtained routinely during the first two days of hospitalization), since they are important for atherosclerotic arterial remodeling.

### Statistical Analysis

Statistical analyses were performed using STATISTICA data analysis software, version 12.0 (StatSoft, Inc. 2008).

The Mann–Whitney U test was used to compare the levels of cells between the stroke and the control groups. The chi-squared test was used to compare risk factors between the patients and the control subjects. Friedman ANOVA and Kendall concordance tests were used to compare levels of the subgroups of cells between days 1, 2, and 7. The Mann–Whitney U test, Spearman rank, and Pearson correlation tests were used to identify differences and correlations between cell numbers and laboratory parameters and neurological or functional scale results. A probability value p < 0.05 was considered statistically significant.

## Results

Co-morbidities and characteristics of the stroke and the control group are presented in Table 1.

On admission, median NIHSS score was 5 (range 1–23), and on day 9, it was 2 (range 0–11). Median pre-mRS score on admission (pre-existing disability) was 0 (range 0–2), and on day 9, it was 2 (range 0–4).

### Stem Cells Levels in the Patients and the Control Group

There were statistically significant differences between the patients and the control group in case of the CD45–CD34+ CXCR7+ and the CD45–CD34 + CD271+ cells.

The patients had lower MFI values of the CD45–CD34 + CXCR7+ cells on day 2 (*p* = 0.028; mean ± SE, patients:0.29 ± 0.08 vs. controls: 0.32 ± 0.04) and on day 7 (*p* = 0.021; mean ± SE, patients: 0.2 ± 0.03 vs. controls:0.32 ± 0.04).

MFI values of the CD45–CD34 + CD271+ cells were lower in the patients on day 1 (*p* = 0.01, mean ± SE, patients: 0.063 ± 0.01 vs. controls: 0.12 ± 0.01), on day 2 (*p* = 0.002; mean ± SE, patients: 0.07 ± 0.025 vs. controls: 0.12 ± 0.01), and on day 7 (*p* = 0.046; mean ± SE, patients: 0.066 ± 0.02 vs. controls: 0.12 ± 0.01).

In the patient group, there were no significant differences in MFI values of the subgroups of cells between days 1, 2, and 7.

**Fig. 1:**
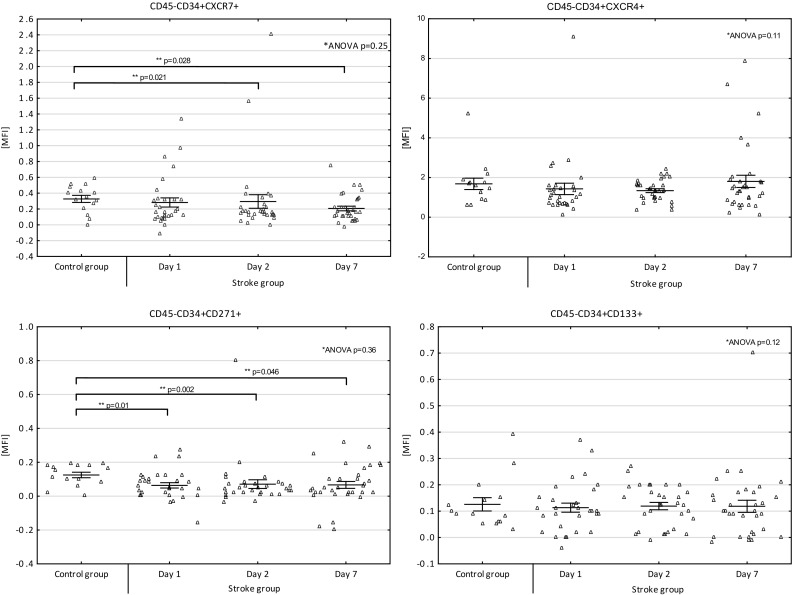
The MFI values of the CD45–CD34 + CXCR7+, CD45–CD34 + CXCR4+, CD45–CD34 + CD271+, CD45–CD34 + CD133+ stem/progenitor cells in the stroke patients (on days 1, 2 and 7) and in the control group (blood was collected once, incidentally). **There were statistically significant differences (Mann–Whitney U test) between the patients and the control group in case of MFI values of the CD45–CD34 + CXCR7+ cells on day 2 and 7, and the CD45–CD34 + CD271+ cells on day 1, 2 and 7, with lower values being present in the patients in all cases.*In the patient group, Friedman ANOVA and Kendall concordance tests were used to compare MFI values of the subgroups of cells between the days 1, 2, and 7—the differences were not statistically significant. Data are presented as individual values (*Δ*) and mean ± SE

The MFI values of the cells in the stroke patients and in the control subjects are presented in Fig. 1.

### Stem Cells and Co-morbidities

Certain co-morbidities in the patients seem to have influenced the levels of some of the cells subgroups. The statistically significant differences are presented in Table 2. Obesity, insulin-dependent diabetes, and hypertension were associated with higher levels of the CD45–CD34 + CXCR4+ and the CD45–CD34 + CXCR7+ cells. Patients abusing alcohol had lower levels of the CD45–CD34 + CD271+ cells.

**Table 2:** Statistically significant differences in MFI values of the stem cells between the stroke patients with or without specific cerebrovascular risk factors

Co-morbidities present (+) or absent (−) in the anamnesis	MFI values of the stem cells [mean ± SD]		*p* value^*^
Hypertension	(+)	(−)	
CD45–CD 34 + CXCR7+ on day 7	0.22 ± 0.17	0.07 ± 0.03	0.045
Alcohol abuse	(+)	(−)	
CD45–CD34 + CD271+ on day 2	0.005 ± 0.023	0.088 ± 0.16	0.031
On day 7	0.015 ± 0.01	0.089 ± 0.12	0.036
Insulin-dependent diabetes	(+)	(−)	
CD45–CD34 + CXCR4+ on day 2	1.915 ± 0.26	1.23 ± 0.53	0.025
Obesity	(+)	(−)	
CD45–CD34 + CXCR4+ on day 1	1.94 ± 2.01	0.88 ± 0.47	0.009

^*^Mann–Whitney U test was used to assess the differences in MFI values of the stem cells between the patients with or without specific co-morbidities. All of the presented differences were statistically significant

Patients with or without the cerebrovascular risk factors did not differ in terms of severity of stroke (there were no statistically significant differences between their NIHSS scores on admission).

### Stem Cells and Ischemic Lesion Volume

Mean lesion volume in MRI 1 was 12088.59 ± 18718.59 mm^3^ and in MRI 2 it was 20707.37 ± 45821.51 mm^3^.

**Fig. 2:**
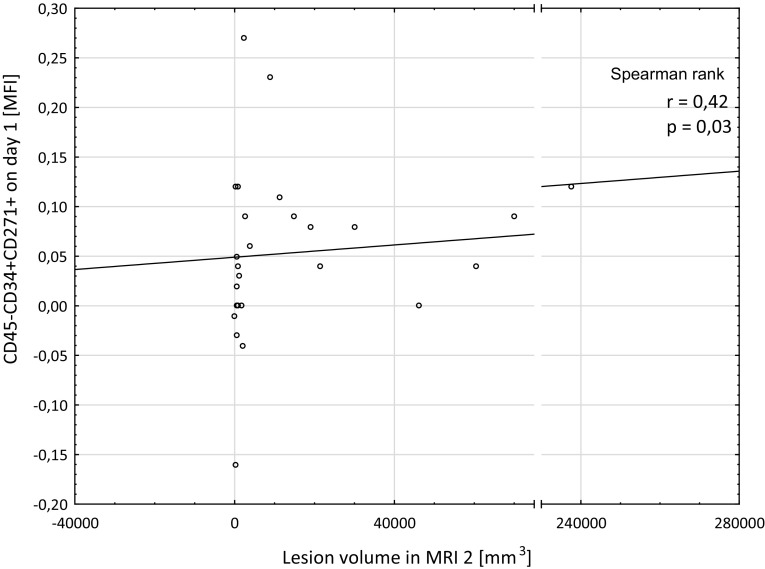
MFI values of the CD45–CD34 + CD271+ cells on day 1 correlated positively with lesion volume in MRI 2 performed on day 5 ± 2 after stroke onset (Spearman rank correlation)

MFI values of the CD45–CD34 + CD271+ cells on day 1 correlated positively with lesion volume in MRI 2 (*r* = 0.42, *p* = 0.03, *n* = 26). This correlation is presented in Fig. 2.

The lesion volume, absolute, and relative differences between the lesion volume in MRI 1 and MRI 2 did not correlate significantly with any other of the cell subgroups.

### Stem Cells and NIHSS Score

MFI values of the CD45–CD34 + CD133+ cells on day 2 correlated negatively with NIHSS score on day 9 (*r* = − 0.36, *p* = 0.04) (Supplementary Fig. 1). There were no other statistically significant correlations between the chosen subgroups of cells and the NIHSS scores on days 1, 2, and 9.

There were no statistically significant differences in the levels of the chosen subgroups of the stem/progenitor cells between the OCSP or TOAST subgroups. Neither did the levels of the cells correlate with mRS scores on day 9.

### Stem Cells and Thrombolytic Treatment

We have found statistically significant differences between the patients who received and those who did not receive the thrombolytic treatment for the CD45–CD34 + CXCR7+ cells on day 1, with higher MFI values being present in the patients that received the treatment (mean ± SE, treated: 0.53 ± 0.14 vs. non-treated: 0.17 ± 0.03, *p* = 0.02). It is worth mentioning that the levels of the cells on day 1 were assessed after the thrombolysis. The patients who underwent thrombolysis did not differ significantly from those without this treatment in terms of NIHSS scores and co-morbidities. Interestingly, MFI values of stem/progenitor cells of the patients who received rtPA did not differ significantly from MFI values of the control subjects.

### Stem Cells and Arterial Stiffness

Mean PWV on day 5 was 11.5 ± 3.2 m/s [mean ± SD], median was 11.2 m/s.

There was a significant negative correlation between the mean PWV on day 5 and MFI values of the CD45–CD34 + CD133+ cells on day 2 (*r* = − 0.47, *p* = 0.034) (Supplementary Fig. 2).

Fifteen patients had PWV > 10 m/s, 13 patients had PVW < 10 m/s, in five patients data were unobtainable due to technical reasons. The patients whose PWV was above 10 m/s had significantly higher MFI values of the CD45–CD34 + CXCR4+ and the CD45–CD34 + CXCR7+ cells on day 1 than those with the value below 10 m/s (CXCR4+ cells: mean ± SE, PWV > 10 m/s: 1.81 ± 0.57 vs. PWV < 10 m/s: 0.94 ± 0.21, *p* = 0.04; CXCR7+ cells: mean ± SE, PWV > 10 m/s: 0.38 ± 0.1 vs. PWV < 10 m/s: 0.13 ± 0.04, *p* = 0.045).

### Stem Cells and Laboratory Parameters

Statistically significant correlations between the stem cells levels and the laboratory parameters are presented in Table 3. The levels of the CD45–CD34 + CXCR7+ and CD45–CD34 + CD271+ cells, both on day 7, correlated positively with the inflammatory parameter—the highest CRP (Supplementary Fig. 3). The highest CRP was 16.07 ± 24.67 mg/dl and was reported on day 4.2 ± 2.6, while our patients were hospitalized for 10 ± 3.9 days [mean ± SD].

**Table 3:** The statistically significant correlations between the stem/progenitor cells MFI levels and the chosen laboratory parameters

	HDL	LDL	Highest CRP
CD45–CD34 + CD133+			
On day 7	*r* = 0.5 *p* = 0.0031^*^		
CD45–CD34 + CXCR7+			
On day 2		*r* = 0.36 *p* = 0.048^*^	
On day 7			*r* = 0.41 *p* = 0.015^*^
CD45–CD34 + CD271+			
On day 2		*r* = 0.55 *p* = 0.001^*^	
On day 7		*r* = 0.41 *p* = 0.019^*^	*r* = 0.36 *p* = 0.038^*^

*HDL* high-density lipoprotein; *LDL* low-density lipoprotein; *highest CRP* the highest value of the C-reactive protein during hospitalization

^*^Spearman rank correlation test was used to assess correlations between the stem cells levels and the laboratory parameters. The table presents only statistically significant correlations

Also, both groups of cells correlated positively with the concentrations of LDL. Additionally, the CD45–CD34 + CD133+ cells correlated positively with HDL values (Supplementary Fig. 4).

Other laboratory parameters chosen in this study did not correlate significantly with the subgroups of stem/progenitor cells.

## Discussion

The findings concerning the CD45–CD34 + CD271+ cells are most intriguing. There have been no studies on this group of cells in acute ischemic stroke patients so far. In our study, the levels of these cells were significantly lower in the patients compared to the control group on all the sampling days. Additionally, the levels of the cells on day 1 correlated significantly and positively with the lesion volume in MRI 2. These results could be a trace of response of this group of cells to the mediators released from the ischemic niche and higher take-up of the cells by the fighting brain. These findings could be important since CD271+ (p75NTR) was initially described to be expressed on the cells of the central and peripheral nervous system and was suggested to be involved in the development, survival, and differentiation of cells. However, it is also a surface marker of mesenchymal progenitor cells with high proliferative, clonogenic, and multipotential differentiation ability (Quirici et al. 2010). Moreover, in a recent study, Paczkowska et al. (2016), reported that bone marrow CD34+ cells spontaneously express neurotrophin receptors (TrkA, TrkB, and TrkC) and the receptor p75NTR (CD271) at higher levels than bone marrow nucleated cells. Moreover, it was reported that even in the absence of inducing factors, the CD34+ cells spontaneously express neurotrophins such as NGF, BDNF, NT-3, and NT-4. Source, availability, and methods of isolation of stem/progenitor cells can be limiting factors for stroke therapies. Bone marrow is the most accessible and abundant source of stem/progenitor cells. If the beneficial effect of the CD45–CD34 + CD271+ cells on ischemic brain is proved, this may be really good news for the regenerative stroke therapies. We assume that optimal timing for the therapy would be the moment of the highest levels of the endogenous CD45–CD34 + CD271+ cells observed in the peripheral blood of stroke patients, which could reflect the increased release of the cells from bone marrow to peripheral blood in response to ischemic lesion mediators. We further hypothesize that “the call” from the brain can be insufficient for the optimal amount of cells to be released from the marrow. Thus, it seems plausible, that in order to enhance the endogenous regenerative potential, the cell/secretome therapy should be applied on the top of “the call” from the brain. We could not prove significant changes of the levels of cells between the days, probably because of the relative mildness of stroke in our patients (the median NIHSS score on admission was 5). However, the highest levels of the CD45–CD34 + CD271+ cells seemed to be present on day 2 (see Fig. 1), which is a similar result to the one received by Iso et al. (2012), in acute myocardial infarction patients, where the CD45 low/−CD34 + CD271+ cell counts peaked on day 3 and then declined gradually up to day 7. Since immunomagnetic cell separation enables the rapid and gentle sorting of specific cell types (Paczkowska et al. 2016) it seems possible to aspirate autologous bone marrow, select the CD45–CD34 + CD271 + cells by immunomagnetic separation, and deliver them back to a stroke patient in the form of intravenous or intra-arterial administration of the cells or their secretomes, without the need of prior cell culture.

We are the first to report on the CXCR7+ progenitor/stem cells in stroke patients. In a study on cerebral ischemia–reperfusion rat hippocampus model, Wang et al. (2014) reported that the over-expressed CXCR7 receptor promoted migration of mesenchymal stem cells towards SDF-1 gradient, acting jointly with the SDF-1/CXCR4 signaling axis. On the other hand, another study on a rat model indicated that ischemic cerebral tissue was infiltrated by the CXCR4+ rather than the CXCR7+ cells (Schonemeier et al. 2008).

Our patients had lower levels of the CD45–CD34 + CXCR7+ cells than subjects from the control group on day 2 and 7. Surprisingly, these differences were not present for the CXCR4+ cells. It was only the CXCR7+ cells that significantly and positively correlated with the inflammatory parameter—the highest CRP. Finally, the patients who received the thrombolytic treatment had higher levels of only the CXCR7+ cells on day 1 (collection performed after the treatment). If we accept the adjuvant role of the CXCR7+ receptor in the SDF-1α–CXCR4 axis, it is surprising that similar correlations for the CD45–CD34 + CXCR4+ cells have not been found. However, there is growing evidence for separate and important functions of CXCR7. There are numerous studies which prove CXCR7 as an independent biomarker of various neoplasms (Demir et al. 2017; Deng et al. 2017; Goto et al. 2017; Gu et al. 2017; Nambara et al. 2017). Furthermore, in a study on coronary arteries of mice and humans, CXCR7 appeared to promote endothelial proliferation and angiogenesis after myocardial infarction (Hao et al. 2017). In another study, knockdown of CXCR7 and not CXCR4, impaired tube formation of endothelial progenitor cells from normal mice, whereas up-regulation of CXCR7 rescued angiogenic function of these cells from diabetic mice (Dai et al. 2017). Finally, isolated knockdown of CXCR7 in human brain microvascular endothelial cells resulted in significantly reduced proliferation, tube formation, and migration of these cells (Liu et al. 2014). Our study also seems to indicate the possibility of the separate CXCR7 function in acute ischemic stroke.

At certain points, however, the two receptors seem to have acted jointly. Both CXCR4+ and CXCR7+ progenitor/stem cells were associated with arterial stiffness. Patients whose PWV values were above 10 m/s had significantly higher levels of the CD45–CD34 + CXCR4+ and the CD45–CD34 + CXCR7+ cells on day 1 than those with the values below 10 m/s. PWV higher than 10 m/s is indicative of worse state of arteries. We hypothesize that higher levels of these cells circulating in the patients with the increased arterial stiffness may be due to the increased inflammatory activity of the chronically injured vessels. There are no stem/progenitor cells studies to confront our results with, but the SDF-1α–CXCR4 axis is reported to have anti-inflammatory and plaque-stabilizing effects (Zernecke et al. 2008) and CXCR7 may be beneficial for atherosclerotic vascular diseases through its cholesterol lowering effect (Li et al. 2014). Interestingly, we have found a positive correlation between the levels of the CD45–CD34 + CXCR7+ cells and LDL values.

Finally, we have also obtained interesting results on the CD45–CD34 + CD133+ cells. There was a significant negative correlation between the mean PWV on day 5 and the levels of the CD45–CD34 + CD133+ cells on day 2. Contrary to our results, Marketou et al. (2014) report a strong positive association between the number of CD45–CD34 + CD133+ cells and PWV in a study on hypertensive patients. This discrepancy may be due to the circumstances of ischemic stroke in our patients.

Only this group of cells correlated with the neurological status of patients. We have received negative correlation with NIHSS score on day 9. Surprising is the lack of correlation with the volume of ischemic lesion reported previously (Bogoslovsky et al. 2010; Taguchi et al. 2004). One of the reasons for the discrepancy could be the choice of surface antigens. The other authors have chosen slightly different surface antigens for characterization of their endothelial progenitor cells, and none of them have chosen the CD45 negative cells. Thus, the comparison is not completely relevant.

Certain co-morbidities seem to have influenced the levels of the cells in the patient group. It may prove that coexistent co-morbidities may influence the process of stem cells efflux from the bone marrow after stroke. Obesity and insulin-dependent diabetes were associated with higher levels of the CD45–CD34 + CXCR4+ cells. There are no human studies to compare our results with, but a mice model study showed opposite associations—baseline levels of circulating CD34 + CXCR4+ cells were decreased and stroke up-regulation of the SDF-1α/CXCR4 axis was reduced in the brain of diabetic mice (Chen et al. 2012). Furthermore, our patients with hypertension had higher levels of the CD45–CD34 + CXCR7+ cells. Interestingly, it has been proved that CXCR7 is important for the re-endothelialization capacity of endothelial progenitor cells in hypertensive patients (Zhang et al. 2014). At the same time, our patients with or without the cerebrovascular risk factors did not differ in terms of severity of stroke (there were no statistically significant differences between their NIHSS scores on admission). Similarly to the case with PWV, it may mean that patients with these risk factors may have higher baseline levels of these cells circulating in response to chronic injury caused by the diseases. However, this hypothesis needs to be proved by comparing the control subjects with and without risk factors, and our control group is too small to enable sensible statistics in this matter. On the contrary, patients abusing alcohol had lower levels of the CD45–CD34 + CD271+ cells. This could indicate specific toxicity of alcohol on this group of cells.

There are some limitations of our study. Firstly, it is a relatively small number of patients included. It was mostly due to the contraindications either for MRI or PWV testing, and due to the time of admission after the incidence of stroke—our patients had to be hospitalized in the first hours after stroke to be included into the study. Secondly, all our patients presented with a relatively mild stroke (on admission the median of the NIHSS score was 5), thus with smaller ischemic lesions, which probably provoked milder pathophysiological reactions, more difficult to be traced by the used methodology. However, our patients had to be healthy enough to be apt for the double MRI and PWV testing. Finally, it would be ideal to present patients who received rtPA as a separate group. Even though the patients who underwent thrombolysis did not differ significantly from those without this treatment in terms of NIHSS scores and co-morbidities, we received some clues that rtPA may influence the levels of circulating stem/progenitor cells. Collecting blood from a bigger group of rtPA patients in future will enable us to perform reasonable statistical analysis in this matter.

## Conclusions

This study discovers possible activity of the CD45–CD34 + CD271+ progenitor/stem cells during the first 7 days after ischemic stroke. It suggests associations of the CD45–CD34 + CD133+ cells with the neurological status of stroke patients, and some activity of the CD45–CD34 + CD133+, the CD45–CD34 + CXCR4+, and the CD45–CD34 + CXCR7+ progenitor/stem cells in the process of arterial remodeling.

## Electronic supplementary material

Below is the link to the electronic supplementary material.

Supplementary material 1 (DOCX 14 KB)

Supplementary material 2 (DOCX 15 KB)

Supplementary material 3 (DOCX 21 KB)

Supplementary material 4 (DOCX 98 KB)
