# Supplementary material for: CD271+, CXCR7+, CXCR4+, and CD133+ Stem/Progenitor Cells and Clinical Characteristics of Acute Ischemic Stroke Patients
Source: Neuromolecular Med. 2018 May 9;20(3):301–11. doi: 10.1007/s12017-018-8494-x (PMC6097064; doi:10.1007/s12017-018-8494-x)
Supplement: Supplementary file 1 — Supplementary material 1 (DOCX 14 KB) [file 12017_2018_8494_MOESM1_ESM.docx]

-0 .05

0 .00

0 .05

0 .10

0 .15

0 .20

0 .25

0 .30

CD45-CD34+CD133+ on day 2 [MFI]

-2

0

2

4

6

8

10

12

NIHSS on day 9 [points]

Spearman rank

r = -0.36

p = 0.04

**Suppl. Fig.1** MFI values of the CD45-CD34+CD133+ cells on day 2 correlated negatively with NIHSS score on day 9 (Spearman rank correlation).
