# Supplementary material for: CD271+, CXCR7+, CXCR4+, and CD133+ Stem/Progenitor Cells and Clinical Characteristics of Acute Ischemic Stroke Patients
Source: Neuromolecular Med. 2018 May 9;20(3):301–11. doi: 10.1007/s12017-018-8494-x (PMC6097064; doi:10.1007/s12017-018-8494-x)
Supplement: Supplementary file 2 — Supplementary material 2 (DOCX 15 KB) [file 12017_2018_8494_MOESM2_ESM.docx]

-0 .05

0

0 .05

0 .1

0 .15

0 .2

0 .25

0 .3

CD45-CD34+CD133+ on day 2 [MFI]

4

6

8

10

12

14

16

18

PWV on day 5 [m/s]

Pearson correlation

r = -0.47

p=0.034

**Suppl. Fig. 2** Pulse wave velocity (PWV) on day 5 correlated negatively with MFI values of the CD45-CD34+CD133+ cells on day 2 (Pearson correlation test).
