# Supplementary material for: CD271+, CXCR7+, CXCR4+, and CD133+ Stem/Progenitor Cells and Clinical Characteristics of Acute Ischemic Stroke Patients
Source: Neuromolecular Med. 2018 May 9;20(3):301–11. doi: 10.1007/s12017-018-8494-x (PMC6097064; doi:10.1007/s12017-018-8494-x)
Supplement: Supplementary file 3 — Supplementary material 3 (DOCX 21 KB) [file 12017_2018_8494_MOESM3_ESM.docx]

Spearman rank

r = 0.36

p = 0.038

B

highest CRP [mg/dl]

-0 .3

-0 .2

-0 .1

0

0.1

0.2

0 .3

0 .4

-20

0

20

40

60

80

100

120

highest CRP [mg/dl]

CD45-CD34+CD271+ cells on day 7 [MFI] [MFI]

-0 .1

0

0 .1

0 .2

0 .3

0 .4

0 .5

0 .6

0 .7

0 .8

-20

0

20

40

60

80

100

120

highest CRP [mg/dl]

CD45-CD34+CXCR7+ on day 7 [MFI]

**A**

Spearman rank

r = 0.41

p = 0.015

Spe

**Suppl. Fig. 3** MFI levels of the CD45-CD34+CXCR7+ (A) and the CD45-CD34+CD271+ (B) cells, both on day 7, correlated positively with the inflammatory parameter – the highest CRP. The highest CRP was 16.07±24.67mg/dl and was reported on day 4.2± 2.6, while our patients were hospitalized for 10 ± 3.9 days [mean±SD]. Spearman rank correlation test was used to assess correlations between the stem cells levels and the CRP values.
