# Supplementary material for: CD271+, CXCR7+, CXCR4+, and CD133+ Stem/Progenitor Cells and Clinical Characteristics of Acute Ischemic Stroke Patients
Source: Neuromolecular Med. 2018 May 9;20(3):301–11. doi: 10.1007/s12017-018-8494-x (PMC6097064; doi:10.1007/s12017-018-8494-x)
Supplement: Supplementary file 4 — Supplementary material 4 (DOCX 98 KB) [file 12017_2018_8494_MOESM4_ESM.docx]

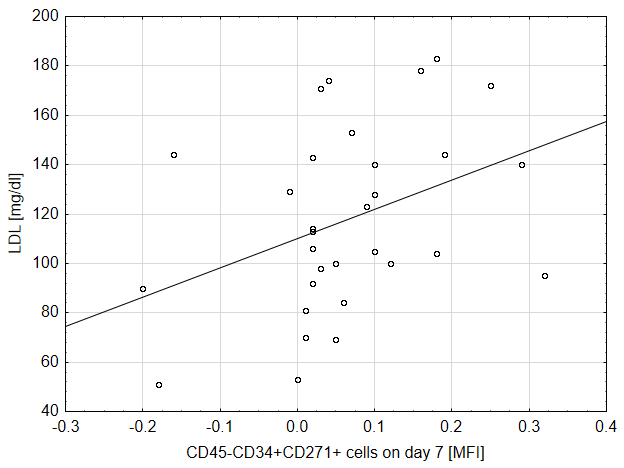


**C**

**D**

Spearman rank

r = 0.36

p = 0.048

Spearman rank

r = 0.5

p= 0.0031

**B**

**A**


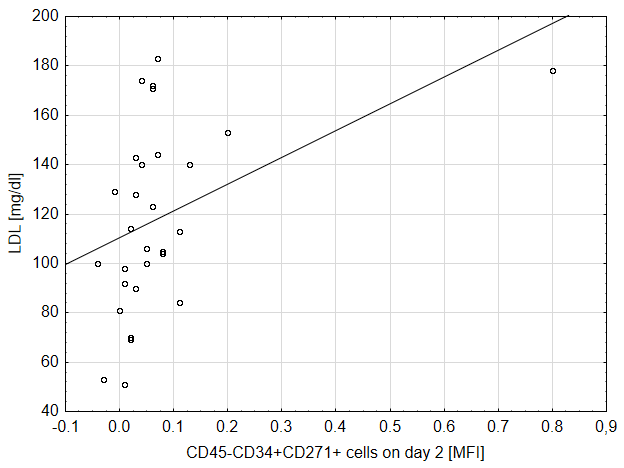

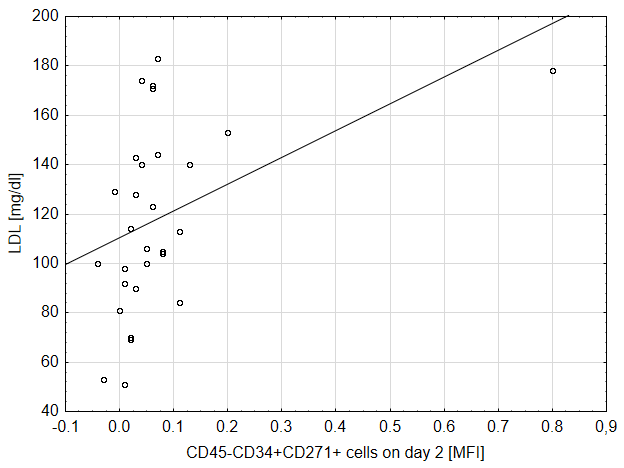

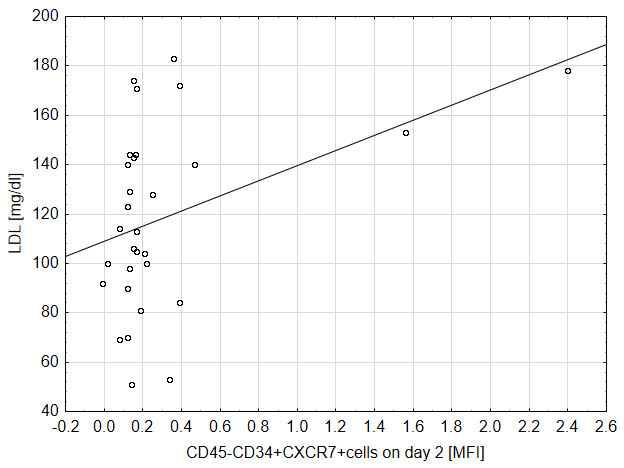

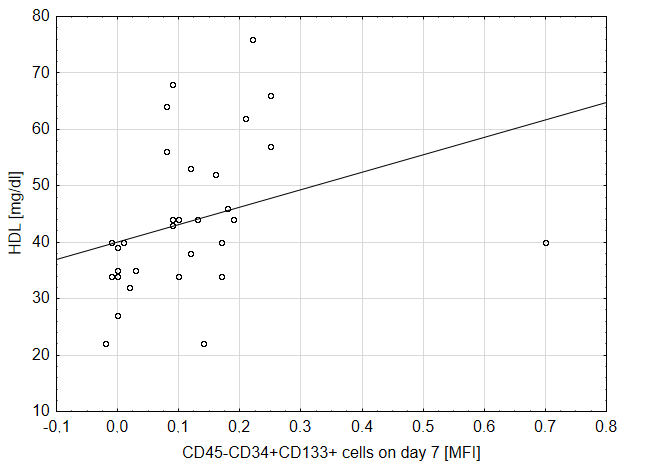


Spearman rank

r = 0.41

p= 0.019

Spearman rank

r = 0.55

p= 0.001

**Suppl. Fig. 4** MFI levels of the CD45-CD34+CD133+ cells on day 7 correlated positively with high-density lipoprotein values (HDL) (A). Low-density lipoprotein (LDL) values correlated positively with MFI values of the CD45-CD34+CXCR7+ cells on day 2 (B) and with the CD45-CD34+CD271+ cells on day 2 (C) and 7 (D). Spearman rank correlation test was used to assess correlations between the stem cells levels and these laboratory parameters.
